# Estimation of Resting Energy Expenditure in Patients Undergoing Total or Partial Pancreatectomy for Pancreatic Tumors

**DOI:** 10.3390/nu18142216

**Published:** 2026-07-08

**Authors:** Pantelis Papanastasiou, Zoe Bouloubasi, Dimitrios Karayiannis, Olga Georgolopoulou, Dimitrios Chasiotis, Ioannis Goulis, Maria Dimitriou

**Affiliations:** 1 Department of Clinical Nutrition, Evaggelismos General Hospital, 10676 Athens, Greece; zoippp@yahoo.com (Z.B.); jimkar_d@yahoo.com (D.K.); olga.geo01@gmail.com (O.G.); 2 Department of Nutritional Science and Dietetics, School of Health Sciences, University of Peloponnese, 24100 Kalamata, Greece; m.dimitriou@go.uop.gr; 3 Laboratory of Hygiene, Social and Preventive Medicine and Medical Statistics, Faculty of Health Sciences, School of Medicine, Aristotle University of Thessaloniki, 54124 Thessaloniki, Greece; 4 2nd Department of Surgery, Evaggelismos General Hospital, 10676 Athens, Greece; dimitrischasiotis@yahoo.gr; 5 Fourth Department of Internal Medicine, Aristotle University of Thessaloniki, Hippokratio General Hospital, 54124 Thessaloniki, Greece; igoulis@auth.gr

**Keywords:** pancreatectomy, pancreaticoduodenectomy, Whipple procedure, indirect calorimetry, resting energy expenditure

## Abstract

Background/Objectives: Total or partial pancreatectomy is associated with significant metabolic stress and high risk of postoperative malnutrition. Accurate estimation of resting energy expenditure (REE) is essential, as predictive equations may not reflect true energy needs. Methods: A prospective study among patients undergoing total or partial pancreatectomy for pancreatic tumors was conducted. REE was measured by indirect calorimetry (mREE) and compared with the Harris–Benedict and Schofield equations and the weight-based approaches (25 and 30 kcal/kg). Agreement was assessed using linear regression and Bland–Altman analysis; accuracy indices included ±10%, Mean Absolute Percentage Error (MAPE) and Root Mean Square Error (RMSE). Results: In 26 patients (mean age, 66.7 ± 8.7 years; 53.8% male) undergoing pancreatic resection (17 pancreaticoduodenectomies, 8 distal pancreatectomies, 1 total pancreatectomy), 60% were at preoperative malnutrition risk. The median measured REE was 1484 kcal/day, rising to 1706 kcal/day after activity adjustment (×1.15) within 14 postoperative days. At 3–6 months postoperatively, patients demonstrated significant declines in nutritional status with a median body weight reduction of −7.3% and a decrease in BMI of −2 kg/m^2^. The 30 kcal/kg method showed the lowest accuracy (MAPE 23.2%, RMSE 416 kcal/day) and overestimated energy needs. Harris–Benedict underestimated mREE in 61.5% of cases, while the 25 kcal/kg approach showed more balanced performance. Conclusions: Postoperative energy expenditure in patients undergoing pancreatic resection appeared elevated relative to predictive equations. Predictive equations lack reliability, favoring indirect calorimetry for precision. Sustained weight loss underscores the need for prolonged nutritional surveillance.

## 1. Introduction

Pancreatectomy is divided into two major types: partial and total pancreatectomy. Total pancreatectomy leads to complete loss of the endocrine and exocrine function of the pancreas, with significant difficulties in maintaining blood glucose control [1]. Contrarily, partial pancreatectomy preserves the endocrine and exocrine functions of the pancreas and allows better control of glucose levels compared to total pancreatectomy. Therefore, partial pancreatectomy is generally the preferred surgical option, excluding cases of advanced cancer, pancreatic metastases or chronic pancreatitis [2,3].

Partial pancreatectomy is classified into two major subtypes, pancreatoduodenectomy (Whipple procedure) and distal pancreatectomy. The Whipple procedure is the standard surgery for tumors in the head of the pancreas. This procedure has been shown to be effective in treating malignant and some benign conditions, but it causes major changes in the digestive system. Therefore, postoperative nutritional support is essential for patients’ recovery and overall health [4].

Most patients will develop malnutrition due to pancreatic insufficiency, impaired digestion and malabsorption after pancreaticoduodenectomy or total pancreatectomy, and they will have higher risk of postoperative complications [5,6]. It should be noted that malnutrition and elevated energy expenditure, although frequently coexisting in this population, represent conceptually distinct phenomena: malnutrition reflects a deficit in nutritional status arising from reduced intake, malabsorption and tissue depletion, whereas increased resting energy expenditure (REE) reflects the metabolic response of the body to surgical stress and systemic inflammation [5,7]. Although there are no specific guidelines for the nutritional management of these patients, the general European Society for Clinical Nutrition and Metabolism (ESPEN) recommendations for cancer patients suggest an energy intake of 25–30 kcal/kg BW/day and a protein intake of 1.0–1.5 g/kg BW/day [8].

Supportive care for patients undergoing total or partial pancreatectomy should incorporate nutritional care as a core component rather than an adjunct to surgical treatment. This population enters surgery with a high preoperative prevalence of malnutrition risk, undergoes a procedure associated with substantial metabolic stress, and frequently develops exocrine and/or endocrine insufficiency postoperatively [9,10,11]. When nutritional needs are not adequately met, the consequences extend beyond weight loss alone, encompassing delayed wound healing, impaired functional recovery, increased postoperative complications, prolonged hospitalization and reduced quality of life. Despite this, no procedure-specific nutritional guidelines currently exist for patients undergoing pancreatic resection, and clinical practice in this area remains inconsistent across institutions. These considerations underscore the rationale for systematically characterizing the energy requirements of this population as a basis for individualized nutritional care.

Surgical stress and the postoperative inflammatory response increase measured energy expenditure (mREE), which requires nutritional support for adequate healing and functional recovery, especially when the patient is malnourished and the inflammatory response is prolonged [8,12]. Although most studies demonstrate that surgical patients enter a hypermetabolic state [13], data on energy metabolism following pancreatectomy remain limited. A previous study examined the association between the Whipple procedure and mREE; however, the small sample size (12 patients) did not draw clear conclusions, highlighting the need for further research in larger populations [13].

Nutritional support following pancreatectomy is a cornerstone of postoperative care, as patients are at high risk of malnutrition due to reduced oral intake, exocrine and endocrine pancreatic insufficiency, metabolic disturbances and increased energy requirements [14,15]. The primary aims of nutritional intervention are to meet energy and protein needs adequately, prevent or mitigate weight and skeletal muscle loss, promote recovery, reduce postoperative complications and improve overall quality of life.

However, the postoperative nutritional management of patients undergoing pancreatectomy varies considerably worldwide, despite its importance. Evidence suggests that less than two thirds of clinical units follow local feeding protocols [14], leading to inconsistencies in care, suboptimal nutritional interventions, delayed recovery and increased risk of complications. Thus, a comprehensive nutritional assessment and energy needs evaluation with systematic and ongoing monitoring is essential to optimize patient outcomes. Standardized evidence-based protocols for nutritional support, when developed and implemented, may standardize clinical practice, improve prognosis, reduce postoperative morbidity and provide the best possible quality of life for patients after total or partial pancreatectomy [16].

This study aimed to determine the mREE of patients undergoing total or partial pancreatectomy using indirect calorimetry and to conduct a comparative evaluation of mREE using commonly used estimation equations or simplified approaches for calculating energy requirements.

## 2. Materials and Methods

### 2.1. Ethical Approval

Data were collected from subjects enrolled between May 2025 and February 2026 at the Surgery Unit of Evangelismos General Hospital of Athens. It was approved by the Institutional Review Board of Evangelismos Hospital of Athens (Approval No. 484, 2025) and the Ethics Committee of the Aristotle University of Thessaloniki (Protocol No. 126/2025) and was conducted in accordance with the ethical principles of the Declaration of Helsinki. The patients were informed about the purpose of the study and consent forms were signed.

### 2.2. Study Population

Inclusion criteria included patients over the age of 18 undergoing total or partial pancreatectomy for pancreatic tumors. Exclusion criteria included age <18 years, pregnancy, and inability to breathe into the indirect calorimetry mask for the duration required for the measurement.

### 2.3. Study Design

This was a prospective observational study. mREE was determined by a clinical dietitian using the Cosmed Q-NRG+ portable indirect calorimetry system. The device was calibrated before each measurement session according to the manufacturer’s instructions using the integrated automatic gas calibration procedure. The measurement was performed using a canopy; in cases where this is not feasible, a face mask is used to enable the recording of VO_2_ and VCO_2_ [17]. Patients were measured in a supine position, awake and motionless, after a minimum of 20–30 min of rest to ensure a steady physiological rate; the first 5 min of each recording were excluded from analysis, and steady state was defined as a period of at least 5 consecutive minutes during which the coefficient of variation in VO_2_ and VCO_2_ did not exceed 10%. Measurements were conducted up to 14 days postoperatively, in the morning at rest, following an 8–10 h fast; they lasted approximately 10 min and were performed at a controlled room temperature (22–24 °C), in accordance with the ESPEN guidelines for measuring REE. The exact day of measurement was determined by clinical stability and the patient’s readiness to undergo the procedure rather than a fixed postoperative day and therefore varied between patients. Patients were also asked to avoid caffeine and strenuous physical activity for at least 12 h prior to the measurement, and supplemental oxygen therapy was temporarily suspended or accounted for, where clinically feasible, during the measurement period.

Anthropometric measurements included body weight, which were recorded using a calibrated electronic scale, Tanita RD-953, to the nearest 0.1 kg, in accordance with standardized procedures. Body weight was measured at three time points: during preoperative evaluation, at discharge, and 3–6 months after discharge. BMI at all three time points was calculated as the quotient of body weight in kilograms divided by the square of height in meters (kg/m^2^) [18]. For height measurement, patients removed any hats and footwear and had their hair unstyled. Patients were asked to stand upright with relaxed hands, pressing their whole foot against the base and resting their heels against the back. Attention was paid to the alignment of the patient’s head using the Frankfort method so that their eyes were level with their upper earlobes without leaning back. The height was measured by lowering the sliding plate to reach the top of the patient’s head. Additionally, the percentage of body weight loss over the last 3 and 6 months was recorded. Five patients could not be reached 3–6 months after discharge.

Biochemical parameters were determined using blood samples collected by the hospital laboratory before and after the mREE measurement and include the measurement of C-reactive protein (CRP) and serum proteins (albumin). Albumin and CRP were included as exploratory variables based on their established roles as markers of nutritional status and systemic inflammation, respectively, both of which have been hypothesized to influence REE in cancer and surgical populations; albumin was used as a surrogate marker of visceral protein stores rather than as a proxy for lean body mass. The analyses were performed in accordance with the hospital’s established standard procedures.

Clinical data were collected from patients’ medical records and include postoperative complications, length of hospital stay, and any readmissions. Prior to surgery, all patients underwent a nutritional risk assessment using the Perioperative Nutrition Screen (PONS) [19], a weighted tool based on four main areas: recent weight loss, reduced food intake or the presence of anorexia, low BMI, and the presence of an underlying disease with high metabolic risk.

### 2.4. Primary and Secondary Outcomes

The main outcomes of the study included mREE, as determined by indirect calorimetry, as well as the percentage of patients’ energy needs met in relation to both the measured mREE and current guidelines.

mREE was calculated from the VO_2_ and VCO_2_ values using the Weir equation [20]: mREE = (3.94 × VO_2_ + 1.11 × VCO_2_) × 1.44. The Respiratory Quotient (RQ) was calculated as the ratio of VCO_2_ to VO_2_. Subsequently, mREE was compared with estimated predicted resting energy expenditure (pREE) values, which were calculated using the Harris–Benedict [21] and Schofield [22] equations.

The Harris–Benedict equation was applied as follows: for men, pREE = 66.47 + 13.75 × weight (kg) + 5.0 × height (cm) − 6.75 × age (years), and for women, pREE = 665.09 + 9.56 × weight (kg) + 1.84 × height (cm) − 4.67 × age (years). The Schofield equation was calculated based on gender and age group: for men aged 30–59, pREE = 11.472 × weight (kg) + 873.1 and for those aged 60 and older, pREE = 11.711 × weight (kg) + 587.7, while for women aged 30–59, pREE = 8.126 × weight (kg) + 845.6, and for those aged 60 and older, pREE = 9.082 × weight (kg) + 658.5. For the calculation, ideal weight was used for overweight patients and corrected weight [Corrected Weight = Ideal Body Weight + 0.25 × (Actual Weight − Ideal Body Weight)] for obese patients. In addition, a comparison was made of total energy requirements, as estimated by mREE adjusted with an activity/stress factor of 1.15 (mREE × 1.15), with the body weight-based equations proposed by the ESPEN guidelines. Ideal body weight was used for the calculation, adjusted based on BMI, using a BMI value of 22 kg/m^2^ for overweight patients and 24.9 kg/m^2^ for obese patients. Comparisons were made between mREE × 1.15 and 25 kcal/kg of ideal body weight, as well as between mREE × 1.15 and 30 kcal/kg of ideal body weight.

Secondary outcomes included length of hospital stay and the incidence of postoperative complications. In addition, the exploratory association between mREE and selected biochemical markers of nutritional status and inflammation (albumin, CRP) was evaluated. The relationship between PONS score and mREE was examined descriptively; a formal statistical association between PONS score and clinical outcomes could not be robustly assessed given the sample size and is not reported as a confirmed finding.

### 2.5. Statistical Analysis

Statistical analysis was conducted using Jamovi version 2.7.15. All (two-tailed) statistical analyses were conducted with a significance level of α = 0.05 and the null hypothesis rejected.

Continuous variables with normal distribution are presented as mean (±standard deviation) and continuous variables with non-normal distribution as median (interquartile range). Qualitative variables are expressed as *n* and relative frequency [*n* (%)]. The distribution normality of all variables was assessed using the Shapiro–Wilk test. For quantitative variables in three groups, Repeated Measures ANOVA was used for variables with normal distribution and Friedman’s test was used for variables without normal distribution.

To further explore the relationship between body weight of patients and mREE, adjusting for other confounding factors, multiple linear regression models were developed. Bland–Altman analysis was performed to evaluate the agreement between the two measurement methods calculated as pREE − mREE for the Harris–Benedict and Schofield equations and as predicted total energy requirement (mREE × 1.15) for the weight-based approaches, with limits of agreement (LoA) defined as the mean difference ± 1.96 standard deviations, to provide information on the systematic bias and LoA.

Finally, an additional accuracy analysis was conducted by calculating the percentage error for each equation using the formula: % Error = (pREE − mREE)/mREE × 100. The estimates were classified as underestimation (<10%), accuracy (±10), or overestimation (>10%). The percentage of patients in each category was calculated, along with overall error metrics such as Root Mean Square Error (RMSE) and Mean Absolute Percentage Error (MAPE). These metrics give a quantitative measure of the clinical accuracy of the equations and enable a more direct comparison of their performance.

## 3. Results

The total study sample consisted of 26 patients, 46.2% of whom were women, with a mean age of 66.7 ± 8.78 years. The most common site of pancreatic cancer was the head of the pancreas (50.0%), followed by the body of the pancreas (15.4%), unspecified pancreatic tumor location (15.4%), the ampulla of Vater (7.7%), Intraductal Papillary Mucinous Neoplasm (IPMN) (7.7%) and the tail of the pancreas (3.8%). The majority of patients underwent a Whipple procedure (65.4%), while a partial pancreatectomy was performed in 30.8% and a total pancreatectomy in 3.8%. The following Table 1, Table 2 and Table 3 present the patients’ anthropometric and clinical characteristics.

**Figure 1:**
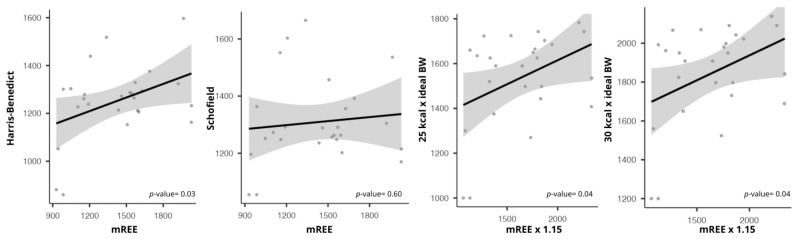
Linear regression between measured resting energy expenditure and a multiplier of 1.15 using energy requirement estimation equations (analyses were performed using mREE-Harris–Benedict; mREE-Schofield; mREE × 1.15 − 25 kcal × ideal body weight; and mREE × 1.15 − 30 kcal × ideal body weight). Data derived from the study’s own prospectively collected patient cohort (*n* = 26).

The linear regression analysis showed that the mREE differed significantly from the Harris–Benedict equation (*p*-value = 0.03) but not from the Schofield equation (*p*-value = 0.60), and it also differed significantly from estimates based on 25 kcal/kg and 30 kcal/kg of ideal body weight (*p*-value = 0.04 for both comparisons) (Figure 1). It should be emphasized that the absence of a statistically significant group-level difference between mREE and the Schofield equation does not imply clinical interchangeability at the individual level, as detailed further below.

**Figure 2:**
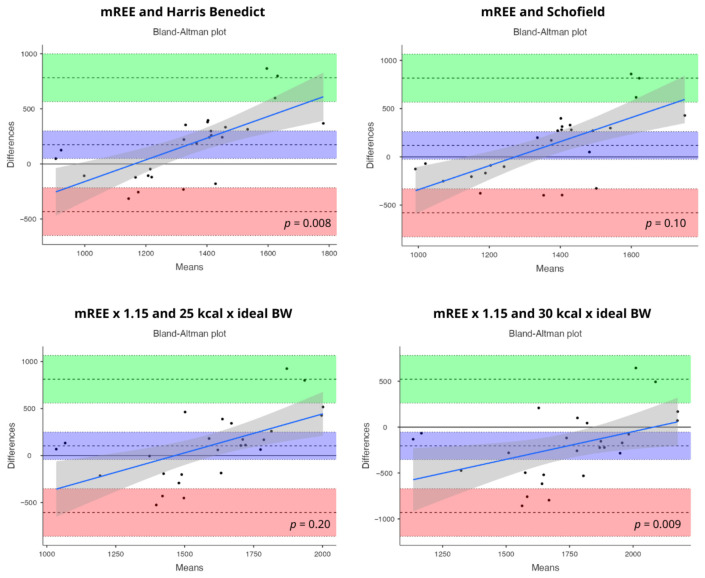
Bland–Altman plots show the limits of agreement between measured resting energy expenditure and predicted resting energy expenditure (Harris–Benedict, Schofield) and between mREE × 1.15 and total energy requirement estimates (25 and 30 kcal/kg of ideal body weight). The dashed lines denote the limits of agreement (mean difference ± 1.96 SD); the solid line denotes the mean difference (bias). The blue shaded region represents the predefined zone of acceptable agreement, whereas the green and red shaded regions indicate predefined thresholds for clinically important overestimation and underestimation, respectively. Data derived from the study’s own prospectively collected patient cohort (*n* = 26).

The Bland–Altman plots showed wide LoA between mREE and all estimated methods, suggesting significant clinical inconsistency at the individual level. Specifically, for the Harris–Benedict equation, the mean difference (bias) was 175 kcal/day (95% CI: 49.5 to 300), with relatively symmetrical but wide LoA of −433 to 782 kcal/day (95% CI for lower limit: −649.7 to −216; 95% CI for upper limit: 565.5 to 999) and an indication of proportional bias (*p*-value = 0.008). Similarly, the Schofield equation showed a smaller mean difference (bias) compared with the previous equation (118 kcal/day, 95% CI: −25.6 to 262, not statistically different from zero), with wide but somewhat larger LoA of −580 to 816 kcal/day (95% CI for lower limit: −828.8 to −331; 95% CI for upper limit: 567.2 to 1065) and no statistically significant proportional bias (*p*-value = 0.10). In contrast, methods based on mREE × 1.15 combined with 25 or 30 kcal/kg of ideal body weight showed greater dispersion and clinically significant deviation: for 25 kcal/kg, the bias was 103 kcal/day (95% CI: −42.9 to 249), with LoA of −606 to 812 kcal/day (95% CI for lower limit: −858.9 to −353; 95% CI for upper limit: 559.3 to 1065) and no significant proportional bias (*p*-value = 0.20); for the 30 kcal/kg approach, the bias was −204 kcal/day (95% CI: −354 to −54.5), with LoA of −930 to 522 kcal/day (95% CI for lower limit: −1189 to −671.3; 95% CI for upper limit: 263 to 781.1) and significant proportional bias (*p*-value = 0.009), with the deviation increasing as the mean values increase. Overall, the wide LoA across all methods demonstrates limited accuracy and interchangeability with mREE (Figure 2).

The analysis presented in Table 4 compares pREE with mREE in 26 patients. Among the resting equations, the Harris–Benedict equation showed the lowest total deviation (RMSE 350 kcal) but the highest percentage of underestimations <10% of mREE (61.5%), and the Schofield equation also showed a high percentage of underestimations <10% of mREE (53.8%). When the body weight-based equations were compared to the adjusted energy requirements (mREE × 1.15), the 25 kcal/kg equation showed moderate accuracy (30.8% within ±10% and 34.6% less than or greater than 10%, RMSE 369 kcal), while the 30 kcal/kg equation showed greater deviation (MAPE 23.2%, RMSE 416 kcal) and lower clinical accuracy, suggesting a tendency to overestimate energy requirements.

Multiple linear regression showed that the model explained 65% of the variance in mREE. Among the variables included in the model (age, sex, CRP, albumin), body weight is an independent predictor of mREE in every model, and for every 1 kg increase in body weight, mREE increases by 8.01 kcal (95% CI: 1.39–14.6, *p*-value = 0.020) (Table 5).

**Figure 3:**
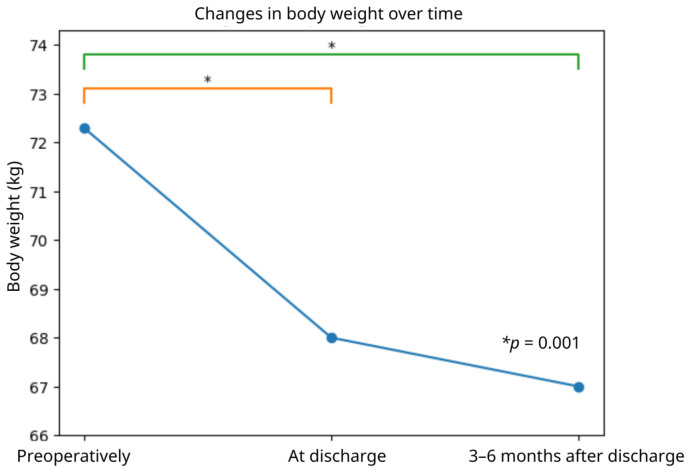
Change in body weight from the preoperative period through discharge and 3–6 months after surgery. Data derived from the study’s own prospectively collected patient cohort (*n* = 26).

**Figure 4:**
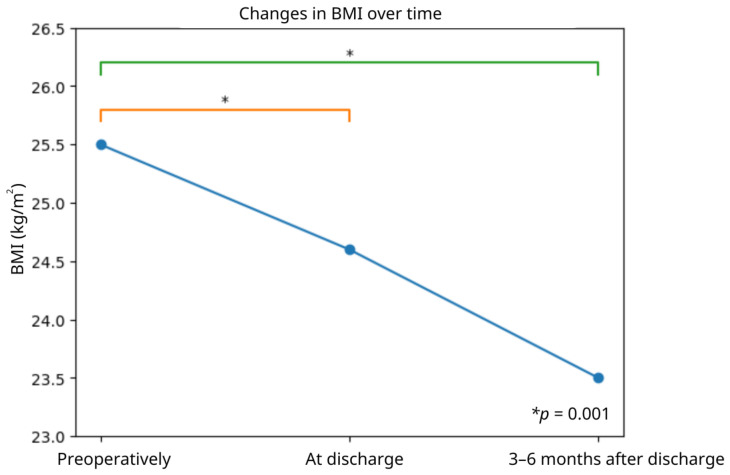
Change in Body Mass Index from the preoperative period through discharge and 3–6 months after surgery. Data derived from the study’s own prospectively collected patient cohort (*n* = 26).

Figure 3 and Figure 4 illustrate the changes in patients’ body weight and BMI from the preoperative period to hospital discharge and the 3–6-month postoperative follow-up. A statistically significant reduction in both body weight and BMI was observed over time (*p* = 0.001). More specifically, body weight decreased significantly by the time of discharge and continued to decline further during the 3–6 months following discharge. A similar pattern was observed for BMI. Pairwise comparisons demonstrated significant differences both between the preoperative period and discharge and between the preoperative period and the 3–6-month follow-up. These findings indicate that the surgical intervention was associated with progressive postoperative weight loss during the first months after surgery.

## 4. Discussion

The aim of this study was to determine mREE in patients undergoing total or partial pancreatectomy, as well as to conduct a comparative assessment of their energy requirements and clinical outcomes. The results showed that mREE amounts to 1484 kcal/day, while with an activity factor of mREE × 1.15, it amounts to 1706 kcal/day up to 14 days postoperatively. The estimation equations showed significant deviations from mREE, while significant postoperative weight loss and a reduction in BMI were also recorded during the first 3–6 months. The patients’ RQ was 0.70 ± 0.09, suggesting greater lipid utilization and mobilization of fat stores and is commonly seen in surgical patients with inadequate caloric intake, prolonged fasting, or underfeeding relative to requirements. This is particularly relevant given the high perioperative prevalence of diabetes mellitus (42.3%) in this cohort, as postoperative hypoinsulinemia and impaired carbohydrate utilization may further favor fat oxidation. Taken together, the low RQ observed in this study may reflect both the metabolic stress of major abdominal surgery and inadequate early postoperative feeding, reinforcing the importance of timely and adequate nutritional support in the immediate postoperative period. However, an RQ at or below 0.70, the theoretical floor for pure fat oxidation, also raises the possibility of measurement artifacts, including air leaks around the canopy or mask, drift in gas-analyzer calibration, or failure to reach a true physiological steady state; this finding should be interpreted with caution. Hypermetabolism is defined as an increase in REE [23]. When evaluated in relation to cancer type, some studies have observed normal REE levels in patients with breast cancer, melanoma, gastric cancer, or rectal cancer [24], while a hypermetabolic state has been observed in patients with pancreatic cancer [13,25]. It should be noted that the tumor-driven hypermetabolism described in the cancer cachexia literature may not directly apply to the present cohort, the majority of whom underwent curative-intent resection of the primary tumor. We therefore interpret the elevated postoperative mREE observed in this study as reflecting predominantly the metabolic response to major abdominal surgery and the accompanying systemic inflammatory response, rather than ongoing tumor-driven hypermetabolism per se. Regarding oncological status during follow-up, only 3-month mortality was systematically recorded in this cohort (one patient, 3.8%) and data on disease recurrence or progression at 3–6 months were not collected.

In contrast, the Harris–Benedict and Schofield equations often show significant deviations from mREE, a finding that has been confirmed in multiple studies involving oncology and surgical patients. In five studies, the results showed that the predictive equations underestimated the mREE of cancer patients, and in three studies, the mREE was overestimated by the predictive equations [26]. In a study with a design similar to the present study, involving 12 patients who underwent the Whipple procedure, the mREE before surgery was 1198 kcal/day and 22.4 kcal/kg/day, while the pREE estimated using the Harris–Benedict equation was 1174 kcal/day and 21.7 kcal/kg/day. Energy expenditure appeared to increase postoperatively, reaching up to 25.4 kcal/kg/day within 14 days after surgery [13]. However, this study did not compare the same predictive equations used in the present study, nor was body weight adjustment performed. It should be noted that the present’s study characterization of postoperative energy expenditure as elevated rests primarily on comparisons with predictive equations and with the findings of the single small prior study of Sasaki. Without preoperative mREE measurements in the same patients it is not possible to definitely establish a hypermetabolic state.

Although a correlation was observed between mREE and the Harris–Benedict equation, the Bland–Altman plots revealed wide LoA, suggesting that, at the individual level, the equations may lead to clinically significant errors. The Harris–Benedict equation demonstrated a significant systematic underestimation of mREE, indicating that its accuracy decreases as energy requirements increase. In contrast, the Schofield equation did not demonstrate proportional bias, suggesting a more stable performance across the range of mREE values. However, the particularly wide LoA indicates substantial interindividual variability. This distinction is clinically important: although the Schofield equation did not differ significantly from mREE at the group level, it nonetheless underestimated mREE by more than 10% in 53.8% of individual patients, with a MAPE of 21.1% and wide LoA on Bland–Altman analysis. Statistical non-significance of a mean difference across the cohort therefore does not equate to acceptable accuracy for any individual patient, and the two types of evidence, group-level comparison and individual-level agreement, should be interpreted as complementary rather than interchangeable. The method based on 30 kcal/kg of ideal body weight showed the greatest deviation, indicating a tendency toward systematic overestimation of energy requirements, especially in patients with higher mREE values. These findings suggest that simplified body weight-based equations may not be appropriate for surgical patients. While indirect calorimetry remains the reference standard for individual-level precision, predictive equations continue to serve an important role in clinical settings where calorimetry is unavailable, and their limitations should be considered when interpreting their results in this patient population.

In the accuracy analyses between pREE equations, the Harris–Benedict equation demonstrated the lowest overall error (RMSE = 350.6 kcal) but also showed the highest rate of mREE underestimation (<10% in 61.5% of cases). The Schofield equation followed with a slightly higher error (RMSE = 368.7 kcal) and greater relative error (MAPE = 21.1%), also demonstrating a significant tendency toward underestimation. In contrast, the weight-based equations compared with the adjusted value of mREE × 1.15 showed a different error distribution: the 25 kcal/kg ideal body weight equation demonstrated the lowest relative error (MAPE = 17.8%) and a more balanced distribution of under- and overestimation, whereas the 30 kcal/kg equation showed the highest overall error (RMSE = 416.8 kcal) and a strong tendency toward overestimation (>10% in 46.2% of cases). These findings further support the position that indirect calorimetry remains the reference method whenever available. Consistent with the findings of the present study, a study evaluating 61 oncology patients prior to surgery reported that the Harris–Benedict equation most frequently underestimated energy expenditure compared with indirect calorimetry, whereas the Schofield equation demonstrated the greatest accuracy among the predictive equations assessed. The reported RMSE values were 914 kcal/day and 794 kcal/day, respectively [27]. Importantly, the Harris–Benedict and Schofield equations were compared against raw mREE, whereas the 25 and 30 kcal/kg approaches were compared against mREE × 1.15; this difference in comparator means the resulting error metrics are not strictly comparable across the two groups of methods, and this distinction should be borne in mind when interpreting the relative accuracy ranking reported above.

From a clinical standpoint, individualized measurement of mREE has direct implications for postoperative nutritional management. Specifically, mREE values can be used to guide the caloric prescription for enteral or parenteral nutrition during the immediate postoperative period, to inform decisions regarding the transition from full nutritional support to oral feeding, and to identify patients at risk of overfeeding as may occur with the 30 kcal/kg approach or underfeeding as may occur with the Harris–Benedict equation. Because weight-based estimates of REE are inherently confounded by the fluid shifts, malabsorption, and catabolism that accompany the early postoperative period, measurement with indirect calorimetry, rather than formula-based estimation, is likely to be of particular clinical value in this population.

Taken together, perhaps the most clinically actionable finding of this study is that the majority of patients were already malnourished before surgery and continued to lose weight for months afterwards. In our cohort, 61.5% of patients screened positive for nutritional risk preoperatively (PONS-positive), and body weight declined progressively and significantly, by a median of 7.3% between the preoperative period and 3–6 months after discharge, with no evidence of weight stabilization by the end of follow-up. It is important to emphasize that malnutrition risk, as captured by the PONS score and mREE, represent distinct constructs: the former reflects the patient’s nutritional state, whereas the latter reflects the metabolic cost of the surgical and inflammatory response, and the two were therefore analyzed as independent outcomes in this study.

A previous study in patients with pancreatic cancer reported significant weight loss and alterations in body composition up to 14 weeks after the Whipple procedure [28]. At 6 months, patients appeared to return to their body weight at admission, although with reduced body composition parameters, a finding that was not observed in the present study. In another study, patients demonstrated significant weight loss and reductions in serum albumin levels, along with a progressive decline in both lean body mass and adipose tissue up to 12 months after the Whipple procedure [29]. In a study analyzing all three major surgical procedures for pancreatic cancer resection, patients who underwent pancreatectomy had lost 8.4% of their body weight 2 months postoperatively and 9% at 4 months after surgery [30]. This degree of weight loss has been significantly associated with poorer postoperative clinical outcomes. Furthermore, an analysis of 1090 patients who underwent pancreatectomy found that the median percentage of weight loss during the first postoperative year was approximately 6.6% (−7.8% for partial pancreatectomy and −4.2% for distal pancreatectomy), with a steady decline in body weight already evident at 1 and 3 months after surgery [31]. In the present study, a statistically significant reduction in body weight was observed both when comparing preoperative values with discharge weight and when comparing preoperative values with body weight at 3–6 months following pancreatectomy. This weight loss likely reflects a combination of reduced food intake, malabsorption and metabolic stress. Moreover, the findings indicate that weight loss is not confined to the immediate postoperative period but may continue for several months after surgery. Consequently, systematic patient follow-up should extend beyond hospitalization, with particular emphasis on the first postoperative months, in order to prevent further deterioration of nutritional status.

Endocrine dysfunction following pancreatic resection also warrants consideration. In the present cohort, 42.3% of patients had preoperative diabetes mellitus, a prevalence that likely reflects tumor-related beta-cell dysfunction prior to surgery. Pancreatogenic diabetes (Type 3c diabetes mellitus) differs metabolically from Type 2 diabetes, being characterized by combined deficiency of both insulin and glucagon secretion, an impaired incretin response, and a tendency toward unpredictable glycemic fluctuations, including a higher risk of hypoglycemia [32]. These alterations may influence substrate utilization and energy metabolism, and postoperative glucose dysregulation represents an additional metabolic burden that should be incorporated into individualized nutritional planning for this patient population.

Beyond elevated energy expenditure, postoperative exocrine pancreatic insufficiency likely represents an important and independent contributor to the progressive weight loss and nutritional deterioration observed in this cohort. Exocrine pancreatic insufficiency is one of the most consequential metabolic complication of pancreatic resection, leading to fat, protein and carbohydrate malabsorption, steatorrhea, and impaired absorption of fat-soluble vitamins, even in patients meeting or exceeding their estimated energy requirements [9,33,34]. In the present study, data on pancreatic enzyme replacement therapy use, the presence of steatorrhea, and objective markers of exocrine function such as fecal elastase-1 were not systematically collected. Consequently, we are unable to determine the relative contributions of hypermetabolism versus malabsorption to the nutritional decline observed after discharge.

Changes in body weight and BMI alone may underestimate clinically meaningful alterations in body composition following pancreatectomy. In the early postoperative period, body weight can be confounded by fluid shifts, third-spacing, and edema, which may temporarily mask or exaggerate true losses of skeletal muscle mass. Postoperative sarcopenia is of particular clinical relevance in this population, having been associated with increased complication risk, reduced tolerance to adjuvant chemotherapy, impaired functional recovery, and poorer quality of life [10,35,36,37]. Because body composition was not assessed in the present study, we were unable to determine the extent to which the observed weight loss reflected losses of fat mass versus lean body mass. We therefore suggest that future studies in this population incorporate objective body composition assessment, such as computed tomography (CT)-derived skeletal muscle measurements or bioelectrical impedance analysis, alongside body weight and BMI. Multiple linear regression analysis demonstrated that body weight was the only independent predictor of mREE. Specifically, for every 1 kg increase in body weight, mREE increased by approximately 8 kcal/day, while other factors, such as CRP (mg/dL) and postoperative albumin levels (g/dL), did not show an independent effect on mREE. This finding is consistent with the literature, where body weight, particularly lean body mass, is considered the main determinant of REE. A meta-analysis of 27 studies reported an increase in REE of 2.31 kcal/kg lean body mass/day in patients with cancer, highlighting the significant contribution of body composition to the regulation of REE [38]. However, it should be noted that certain potentially important factors, such as tumor size, were not included in the present model. It has been suggested that the additional energy expenditure associated with tumor burden may range from 100 to 1400 kcal/day. Theoretically, an additional energy requirement of approximately 400 kcal/day would correspond to nearly 25% of the REE of a patient with a daily REE of 1600 kcal, emphasizing the potentially substantial impact of tumor burden on metabolism [39].

Additionally, length of hospital stay is an important indicator of postoperative recovery and is directly related to nutritional support and patients’ energy requirements. A study involving a large population of patients undergoing major oncologic surgery demonstrated that hospital stay was reduced with the implementation of ERAS protocols, with an average duration of 6 days. However, length of stay was influenced by the type of surgery and postoperative complications, significantly affecting healthcare costs and resource utilization [40]. In another study including 668 patients who underwent the Whipple procedure, the mean postoperative hospital stay was 10 days, and this finding was significantly associated with the severity of complications, suggesting that total hospitalization duration more comprehensively reflects the severity of the postoperative condition [41]. In the present study, the median length of hospital stay was 22 days. It also appeared that greater weight loss occurred during hospitalization compared with the post-discharge period. Prolonged hospitalization may be associated with sustained catabolism, increased energy requirements and greater body weight loss. Therefore, length of stay should not be considered solely an administrative quality indicator, but also a potential indirect marker of metabolic burden.

According to the above findings, an important strength of the present study is the use of indirect calorimetry for the measurement of REE. According to the ESPEN guidelines, indirect calorimetry is considered the preferred method for estimating energy requirements, particularly in patients with increased metabolic stress or a high risk of malnutrition. This study was not limited to a single predictive equation but compared mREE with the Harris–Benedict and Schofield equations, as well as with the simplified approaches of 25 and 30 kcal/kg. In addition, both statistical (regression models) and clinical methods of evaluation (Bland–Altman analysis, MAPE, RMSE and ±10% accuracy) were applied, providing a comprehensive assessment of agreement rather than simple correlation alone. Multiple linear regression further strengthened the interpretation of the findings by identifying body weight as an independent predictor of mREE. Furthermore, the collection of preoperative, postoperative and 3–6-month follow-up data provided a dynamic overview of the metabolic course of patients and documented the progressive weight loss observed after pancreatectomy, a finding of particular importance for the planning of nutritional follow-up. Patients undergoing total or partial pancreatectomy for pancreatic tumors represent a unique population at high risk of malnutrition and metabolic disturbances. The present study contributes to the literature by addressing an area in which available data remain limited. Finally, to our knowledge, this is the first systematic effort in Greece to record and evaluate REE using indirect calorimetry in patients undergoing total or partial pancreatectomy. The comparative analysis with established predictive equations and simplified methods for estimating energy requirements provides primary data for the Greek population and establishes a basis for the development of individualized nutritional protocols in this particularly vulnerable group of patients.

The present study also has several limitations that should be considered when interpreting the findings. First, the relatively small sample size may limit both the statistical power and the generalizability of the results. Although the differences between mREE and the predictive equations were statistically significant, a larger sample could further confirm the consistency of the observed discrepancies. Because the exact postoperative day of measurement varied between patients within the 14-day window, mREE values obtained at different time points may not be directly comparable across patients. REE following major surgery is dynamic and likely changes substantially between the immediate postoperative phase and the recovery period. Therefore, the absence of repeated measurements at additional time points may not fully capture the metabolic trajectory of the patients. We also acknowledge that, for a subset of patients with RQ values at or below the theoretical floor of fat oxidation, measurement conditions including calibration drift and canopy/mask seal may have contributed to the result, representing a potential validity concern in addition to a genuine metabolic finding. Moreover, body composition assessment (e.g., lean body mass) was not performed, despite being a major determinant of REE. Consequently, the observed weight loss could not be accurately differentiated into losses of fat mass or lean body mass, limiting the interpretation of metabolic adaptation. Furthermore, data on pancreatic enzyme replacement therapy, steatorrhea and fecal elastase-1 were not systematically collected, limiting our ability to distinguish the contribution of exocrine pancreatic insufficiency from hypermetabolism to the observed nutritional decline. Additionally, the cohort encompassed tumors with heterogeneous biological behavior, including ductal adenocarcinoma of the head, body and tail, ampullary carcinoma, and premalignant lesions such as IPMN, which likely differ substantially in inflammatory burden and cachexia risk. This heterogeneity may have contributed to the considerable interindividual variability in mREE observed in our study and should be addressed through stratified analyses in larger future cohorts. We also note that the cohort included only a single patient who underwent total pancreatectomy, resulting in complete loss of both endocrine and exocrine pancreatic function, a metabolic profile fundamentally distinct from that of patients undergoing partial resection. Given this small number, a formal subgroup analysis by surgical type was not feasible, and the inclusion of this patient in group-level analyses should be interpreted with appropriate caution. Future studies with larger cohorts should report outcomes separately for total versus partial pancreatectomy. Last but not least, the attrition rate was 20% in the 3–6-month follow-up period, which may lead to follow-up bias and reduce the statistical power of the longitudinal analyses. In addition, several unmeasured patient-related factors, including tumor stage, the presence of infection or sepsis, the use of vasoactive agents, mechanical ventilation, and individual variability in physical activity, may have influenced mREE values and were not accounted for in the present analysis.

Taken together, these findings carry several practical implications for the nutritional care of patients undergoing pancreatic resection. First, individualized energy assessment using indirect calorimetry, where available, should be prioritized over predictive equations given the substantial individual-level error demonstrated in this study. Second, where calorimetry is unavailable, clinicians should remain aware of the systematic biases of commonly used predictive equations and weight-based approaches, applying them with appropriate caution rather than as definitive estimates. Third, nutritional follow-up should extend well beyond hospital discharge, given the sustained weight loss observed up to 3–6 months postoperatively. Fourth, the potential contribution of exocrine pancreatic insufficiency to nutritional decline should prompt consideration of pancreatic enzyme replacement therapy where clinically indicated. Finally, nutritional care should be integrated into the broader oncological management pathway for these patients, alongside surveillance for endocrine dysfunction and, where relevant, coordination with oncological treatment planning.

Despite the limitations, this study provides clinically relevant data regarding the accuracy of predictive equations in patients following total or partial pancreatectomy for pancreatic tumors and highlights the importance of individualized assessment of energy requirements. Future studies should focus on nutritional adequacy, body composition assessment and multicenter study designs.

## 5. Conclusions

The present study demonstrated that patients undergoing total or partial pancreatectomy for pancreatic tumors experience significant metabolic and nutritional alterations during the perioperative and early postoperative period. Notably, the cohort was predominantly composed of patients undergoing pancreaticoduodenectomy (65.4%), with a minority undergoing distal pancreatectomy (30.8%) and only a single patient undergoing total pancreatectomy (3.8%), and the inclusion of ampullary tumors and IPMN further introduces pathological heterogeneity. mREE, as determined by indirect calorimetry, differed significantly from predictive equations (Harris–Benedict and Schofield) as well as from the simplified ESPEN body weight-based approaches (25 and 30 kcal/kg), indicating that these methods are not interchangeable with direct measurement at the individual level. Nevertheless, given the limited availability of indirect calorimetry in many clinical settings, predictive equations and simplified weight-based approaches remain a necessary and practical alternative, provided their inherent limitations are taken into account.

Body weight emerged as the only independent predictor of mREE, with an increase of approximately 8 kcal/day for each additional kilogram of body weight. Notably, the majority of patients already presented with preoperative nutritional risk and weight loss and reduction of BMI continued well beyond hospital discharge without evidence of stabilization at 3–6 months, a trajectory that represents the most clinically actionable finding of this study and reinforces the need for nutritional surveillance protocols extending several months into the recovery period.

Overall, the findings support the use of individualized assessment through indirect calorimetry as the most appropriate approach for determining energy requirements in this vulnerable patient population. The generalizability of these findings, however, is limited by the relatively small sample size and the single-center design of the study, and future multicenter studies with larger cohorts are warranted to confirm and extend these results.

## Figures and Tables

**Table 1:** Descriptive characteristics of patients after total or partial pancreatectomy ^1^.

Variable	*n* = 26
Gender (women, *n*, %)	12 (46.2%)
Age (years)	66.7 (±8.78)
Pancreatic cancer site (*n*, %)	
Ampulla of Vater	2 (7.7%)
Body	4 (15.4%)
Head	13 (50.0%)
Tail	1 (3.8%)
IPMN	2 (7.7%)
Unspecified	4 (15.4%)
Type of surgical procedure (*n*, %)	
Whipple procedure	17 (65.4%)
Distal pancreatectomy	8 (30.8%)
Total pancreatectomy	1 (3.8%)
Comorbidities (*n*, %)	23 (88.5%)
Preoperative diabetes mellitus (*n*, %)	11 (42.3%)
Positive PONS (*n*, %)	16 (61.5%)
Length of hospital stay (days)	22 (17.8)
Incidence of complications (*n*, %)	4 (15.3%)
3-month mortality (*n*, %)	1 (3.8%)

^1^ The values correspond to the mean (±standard deviation) or median (interquartile range) for continuous variables with normal and non-normal distributions, respectively. The values correspond to absolute numbers (%) for categorical variables. Abbreviations: IPMN = Intraductal Papillary Mucinous Neoplasm, PONS = Perioperative Nutrition Screen.

**Table 2:** Nutritional assessment and laboratory parameters of patients ^1^.

Variable	Preoperatively	At Discharge	3–6 Months After Discharge	Within Groups *p*-Value
Body weight (kg)	72.3 (16.5)	68.0 (18.9)	67 (15.0) (*n* = 21)	<0.001 *
BMI (kg/m^2^)	25.5 (5.17)	24.6 (5.85)	23.5 (5.63) (*n* = 21)	<0.001 *
Albumin (g/dL)	3.90 (0.75)	3.10 (0.67)	-	<0.001 *
C-reactive protein (mg/dL)	7.80 (3.45)	5.86 (4.56)	-	<0.001 *

^1^ Body weight and BMI were measured at three time points and compared using Friedman’s test; albumin and CRP were measured at two time points (preoperative and at discharge) and compared using the Wilcoxon signed-rank test. * Indicates a statistically significant difference (Friedman’s test for body weight/BMI; Wilcoxon signed-rank test for albumin/CRP). Abbreviations: BMI = Body Mass Index.

**Table 3:** Measured energy consumption and estimated energy consumption of patients ^1^.

Variable	*n* = 26
mREE (kcal/day)	1484 (444)
RQ	0.70 (0.05)
Harris–Benedict (kcal/day)	1268 (90)
Schofield (kcal/day)	1268 (122)
mREE × 1.15 (kcal/day)	1706 (510)
25 kcal/kg × ideal body weight	1608 (223)
30 kcal/kg × ideal body weight	1929 (269)

^1^ The values correspond to the median (interquartile range) for continuous variables with a non-normal distribution. Abbreviations: mREE = measured resting energy expenditure, RQ = Respiratory Quotient.

**Table 4:** Accuracy metrics of equations estimating energy requirements and measured resting energy expenditure ^1,2^.

Method	Underestimation (<10%)	Accuracy (±10%)	Overestimation (>10%)	MAPE	RMSE
Harris–Benedict	61.5%	11.5%	26.9%	18.9%	350 kcal
Schofield	53.8%	15.4%	30.8%	21.1%	368 kcal
25 kcal/kg	34.6%	30.8%	34.6%	17.8%	369 kcal
30 kcal/kg	19.2%	34.6%	46.2%	23.2%	416 kcal

^1^ Abbreviations: MAPE = Mean Absolute Percentage Error, RMSE = Root Mean Square Error ^2^ Harris–Benedict and Schofield estimate basal/resting energy expenditure and are therefore compared with raw mREE; the 25 and 30 kcal/kg weight-based approaches estimate total energy requirements and are therefore compared with mREE × 1.15 (activity-adjusted). Error metrics (MAPE, RMSE) are not directly comparable between these two groups of methods for this reason. Data derived from the study’s own prospectively collected patient cohort (*n* = 26).

**Table 5:** Association between body weight and measured resting energy expenditure using multiple linear regression models ^1,2^.

Model	β	SE	R^2^	%95 CI	*p*-Value
Model 1	8.98	3.16	0.50	2.45–15.5	0.009 *
Model 2	8.83	3.06	0.60	2.48–15.2	0.009 *
Model 3	8.01	3.16	0.65	1.39–14.6	0.020 *

^1^ Model 1: unadjusted; Model 2: age, sex; Model 3: Model 2 plus CRP (mg/dL) and postoperative albumin (g/dL). ^2^ Preoperative body weight was used as the predictor in all models, as postoperative weight measurements in the early recovery period may be confounded by fluid shifts and edema; this introduces a temporal mismatch between the predictor (preoperative weight) and the outcome (postoperative mREE). * Indicates a statistically significant difference between groups. Abbreviations: SE = Standard Error, CI = Confidence Interval. Data derived from the study’s own prospectively collected patient cohort (*n* = 26).

## Data Availability

The data presented in this study are available on request from the corresponding author (the data are not publicly available due to ethical restrictions).

## Abbreviations

The following abbreviations are used in this manuscript:

BMI	Body Mass Index
CRP	C-reactive protein
ESPEN	European Society for Clinical Nutrition and Metabolism
IPMN	Intraductal Papillary Mucinous Neoplasm
LoA	Limits of agreement
MAPE	Mean Absolute Percentage Error
mREE	Measured Resting Energy Expenditure
PONS	Perioperative Nutrition Screen
pREE	Predicted Resting Energy Expenditure
RMSE	Root Mean Square Error
RQ	Respiratory Quotient
